# A giant adrenal lipoma presenting in a woman with chronic mild postprandial abdominal pain: a case report

**DOI:** 10.1186/1752-1947-5-136

**Published:** 2011-04-05

**Authors:** Stylianos Kapetanakis, Ioannis Drygiannakis, Anastasios Tzortzinis, Nikolaos Papanas, Aliki Fiska

**Affiliations:** 1 Surgical Department, Navy Hospital of Crete, Chania, Greece; 2 Department of Anatomy, Democritus University of Thrace, Alexandroupolis, Greece; 3 Second Department of Internal Medicine, Democritus University of Thrace, Alexandroupolis, Greece

## Abstract

**Introduction:**

Adrenal lipomas are rare, small, benign, non-functioning tumors, which must be histopathologically differentiated from other tumors such as myelolipomas or liposarcomas. They are usually identified incidentally during autopsy, imaging, or laparotomy. Occasionally, they may present acutely due to complications such as abdominal pain from retroperitoneal bleeding, or systemic symptoms of infection. We report a giant adrenal lipoma (to the best of our knowledge, the second largest in the literature) clinically presenting with chronic mild postprandial pain.

**Case presentation:**

A 54-year-old Caucasian woman presented several times over a period of 10 years to various emergency departments complaining of long-term mild postprandial abdominal pain. Although clinical examinations were unrevealing, an abdominal computed tomography scan performed at her most recent presentation led to the identification of a large lipoma of the left adrenal gland, which occupied most of the retroperitoneal space. Myelolipoma was ruled out due to the absence of megakaryocytes, immature leukocytes, or erythrocytes. Liposarcoma was ruled out due to the absence of lipoblasts. The size of the lipoma (16 × 14 × 7 cm) is, to the best of our knowledge, the second largest reported to date. After surgical resection, our patient was relieved of her symptoms and remains healthy six years postoperatively.

**Conclusion:**

Physicians should be aware that differential diagnosis of mild chronic abdominal pain in patients presenting in emergency rooms may include large adrenal lipomas. When initial diagnostic investigation is not revealing, out-patient specialist evaluation should be planned to enable appropriate further investigations.

## Introduction

Adenomas, pheochromocytomas, and adrenocortical carcinomas represent the most common tumors of the adrenal glands [1]. In contrast, lipomatous tumors are rare, comprising 4.8% of all primary adrenal tumors and include myelolipomas, lipomas, angiomyolipomas, and liposarcomas [2,3], myelolipomas being the most common [4]. Adrenal lipomas are uncommon. However, the widespread use of ultrasound and computed tomography (CT) has led to their being increasingly discovered as incidental findings during routine examination. Their overall incidence on autopsy is 6% and 4% in imaging studies [5]. Their frequency tends to increase with age.

A total of 18 cases have been reported since 1966, as summarized in Table 1[2-4,6-14]. In all, 58% were asymptomatic or were found post-mortem [3,6,8,10,11]. However, there are case reports of adrenal lipomas presenting either with acute abdominal pain due to hemorrhage [6] or abscess [9], or with subacute to chronic manifestations, such as hypertension or pain [6,12-14].

**Table 1:** All adrenal lipomas reported to date, except those mentioned in Myśliwiec *et al*. [2] where no other clinical information was provided.

**No**.	Author	Year	Sex	Age, years	Localization	Imaging	Dimensions/weight	Clinical setting
1	Lange [11]	1966	M	54	Right		2.5 cm	Necropsy, paroxysmal hypertension

2	Prinz *et al*. [6]	1982	F	73	Right	CT	3 cm	Incidental finding

3	Avinoach *et al*. [6]	1989	F	40	Right		1.3 cm/7 g	Incidental finding (laparotomy)

4	Abe *et al*. [13]	1994	M	56	Left	US/CT	250 g	Pain

5	Lam *et al*. [3]	1997	F	64	Right	US	8 cm/190 g	Incidental finding/disease free >7 years

6	Lam *et al*. [3]	1997	M	78	Right		4.5 cm/24 g	Incidental finding (necropsy)

7	Ghavamian *et al*. [6]	1998	F	50	Left	CT	8 cm	Incidental finding (CT)

8	Guereirro *et al*. [14]	1998	F	66	Right	US/CT	7 × 6 × 6 cm	Hypertension

9	Sharma *et al*. [12]	1998	M	45	Right		12 × 10 × 5 cm/225 g	Pain, hypertension

10	Lam *et al*. [3]	2001	M	65	Left		2 cm	Incidental finding (necropsy)

11	Butner [6]	2002	M	50	Right		1.1 cm	Incidental finding (necropsy)

12	Milathianakis *et al*. [10]	2002	M	39	Right	US/CT	20 cm	Incidental finding

13	Rodriguez-Calvo *et al*. [8]	2007	M	70	Left		1 cm	Incidental finding (necropsy)

14	Rodriguez-Calvo *et al*. [8]	2007	M	45	Right		2 cm	Incidental finding (necropsy), pheochromocytoma (left)

15	Shumaker *et al*.	2008	M	68	Left	CT/MRI	7 cm/135 g	Hypertension, pain

16	Singaporewalla *et al*.	2009	M	44	Left	CT	15.6 cm	Retroperitoneal bleeding

17	Gupta *et al*. [9]	2009	M	51	Right	CT	11.6 × 14.9 × 14.9 cm	Complicated by perinephric abscess

18	Shah *et al*.	2009	M	35	Right	IVU/US/CT		Pain

19	Our case	2010	F	54	Left	CT	16 × 14 × 7 cm	Pain

CT = computed tomography; US = ultrasound.

We report the first adrenal lipoma presenting as chronic mild postprandial pain, also the second largest among the 19 cases reported thus far [2-4,6-14].

## Case presentation

A 54-year-old Caucasian woman presented to our emergency department complaining of mild postprandial abdominal pain and fullness with a duration of 10 years. Her medical history included hypertension, diabetes mellitus and hypercholesterolemia.

On physical examination, our patient was obese with a body mass index of 31 kg/m^2^. Abdominal inspection was unremarkable. No murmurs were auscultated and abdominal sounds were normal both in frequency and in tone. There was mildly increased tympany on periumbilical percussion with no dullness. Abdominal palpation was unrevealing. The same applied to bimanual examination of both hypochondric areas. The remainder of the physical examination was normal, as were laboratory tests.

**Figure 1:**
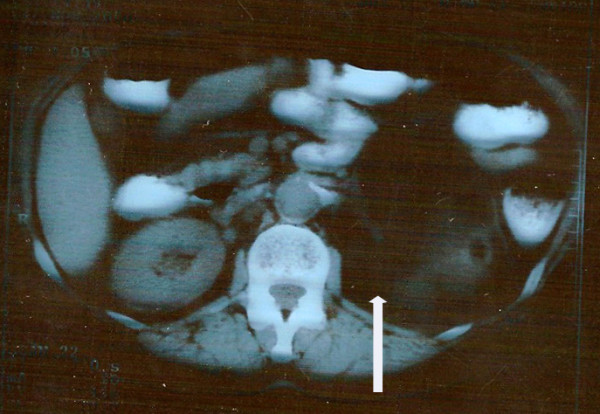
**Computed tomography scan demonstrating a well circumscribed tumor (arrow) in the left retroperitoneal space with adipose tissue density (Hounsfield units = 100) after oral administration of contrast agent**.

An abdominal CT scan was performed with oral administration of contrast. A large retroperitoneal mass (16 × 14 × 7 cm) was found, arising from the left adrenal gland and surrounded by a thin capsule. It exhibited both the density of adipose and solid tissue and was adjacent to but not invading the small intestine, pancreas, spleen, and left kidney. There was no evidence of lymph node or vascular infiltration (Figure 1).

**Figure 2:**
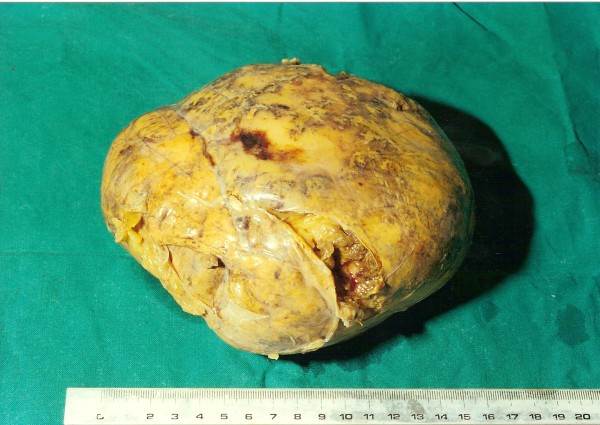
**Macroscopic appearance of the resected tumor**. Tumor dimensions were 16 × 14 × 7 cm with a yellowish smooth external surface.

Laparotomy was performed via a midline incision. After mobilization of the splenic flexure, a large mass was identified arising from the upper pole of the left kidney and involving the left adrenal gland. No obvious lymphadenopathy was seen. The mass was distinct and separate from the surrounding tissues. The entire left adrenal gland containing the tumor was excised (Figure 2).

**Figure 3:**
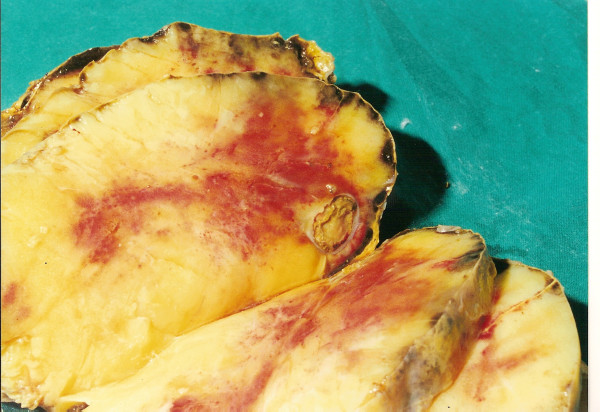
**Transverse section of the resected tumor; homogenous with yellowish- brown color**. The left adrenal gland is identified as a thin layer of orange tissue just below the capsule.

Histological examination revealed a large adrenal lipoma, measuring 16 × 14 × 7 cm and weighing 950 g. Macroscopically, it had a smooth, soft surface (Figure 2). The adrenal gland was recognizable in an area of the external surface. The tumor was partially surrounded by a thin layer of adrenal gland cortex. During dissection, the tumor appeared relatively homogenous and yellowish-brown in color, with occasional irregular white or red-brown lesions (Figure 3).

**Figure 4:**
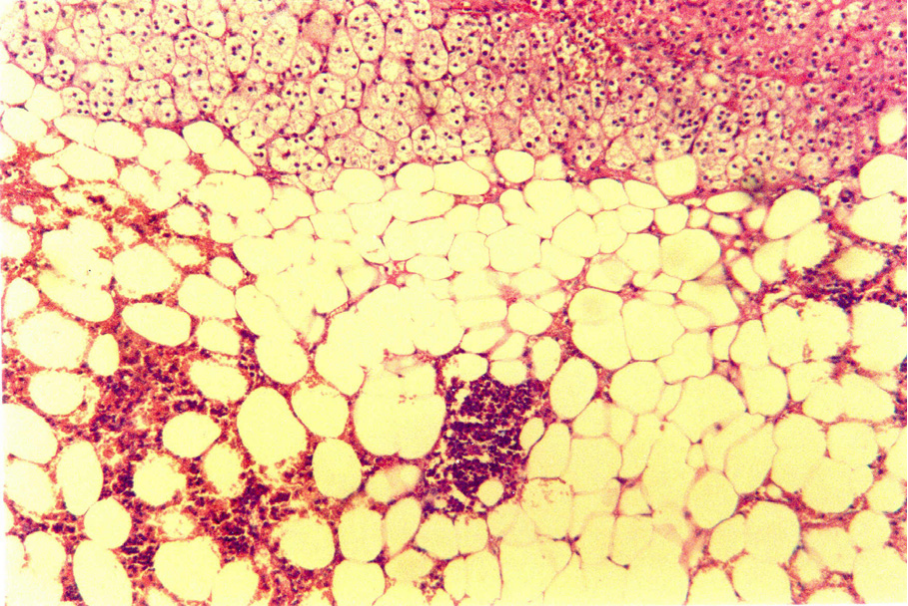
**Histology of adrenal lipoma**. Loci of hemorrhagic infiltrates, liponecrosis, and inflammation, but no immature bone-marrow-derived cells.

Sections (1 cm) were examined by an experienced pathologist. Microscopically, the tumor consisted of large adipocytes without nuclear atypia or mitoses. In several locations, edema or fibrosis by connective tissue with low cell density were observed. Diffuse inflammatory, mostly lymphocytic and occasionally plasmatocytic, infiltrations and occasional depositions of hemosiderin were observed. Small loci of hemorrhage were observed in areas where the cortex was adjacent to the adipose tissue of the tumor. No megakaryocytes were found. Moreover, erythrocytes and leukocytes found were clearly mature, hemorrhagic, with inflammatory infiltrates [15]. Thus, the tumor was diagnosed as a lipoma (Figure 4).

Our patient had an uncomplicated postoperative course and was discharged on the ninth postoperative day in good condition. She became entirely asymptomatic and follow-up was uneventful for six years postoperatively.

## Discussion

Lipomas are benign tumors of uncertain origin that may develop throughout the body. Most originate in the gastrointestinal tract (65% to 75% in the colon). Lipomas of the adrenal gland are very rare [3-5]. The majority of reported adrenal lipomas are small incidentalomas [3,6,8,10,11] and fewer than half are symptomatic (Table 1). Symptoms may be acute, subacute, or chronic. Acute symptoms are rare, such as fever and chills due to complications of perinephric abscess [9] or spontaneous hemorrhage [6]. Subacute or chronic symptoms comprise pain due to large size [6,12,13] and hypertension due to adrenal medullary compression [6,11,12,14].

Histologically, adrenal lipomas consist of bright yellow fat separated by fine fibrous trabeculae. Microscopically, they are composed of mature adipose tissue without cellular atypia. Areas of necrosis, infarct, hemorrhage, inflammatory infiltrates, and calcification may be present. Unlike adrenal myelolipomas, no immature hemopoietic cells exist in adrenal lipomas [15]. It is important to distinguish between histiocytes associated with fat necrosis and lipoblasts. Their frequent arrangement in a circumferential fashion around a large lipid droplet (fat necrosis) is a helpful diagnostic sign. The presence of lipoblasts is the histological hallmark of liposarcoma [2,4].

The rising number of adrenal lipomas reported over the past several years may be attributable to the increasing use of improved imaging techniques [4,6]. CT can easily identify a lipoma by means of its fat content (Hounsfield units between -80 and -120); thus, it is the imaging modality of choice [5]. Lipomas usually present incidentally on CT or ultrasound (Table 1). They are also identified at autopsy but are not the cause of death [3,6,8,11].

There are two case reports in the literature on adrenal lipomas presenting with acute symptoms. The first describes abdominal pain secondary to hemorrhage [6] and the second reports subacute systemic symptoms (fever, chills, and weight loss) for six months [9]. To the best of our knowledge we report the first case in the literature of a patient with an adrenal lipoma clinically presenting with chronic symptoms (10 years) of mild postrandial abdominal pain and fullness, which completely resolved after surgical excision.

In the literature, the diameters of adrenal lipomas range from 1.1 cm to 20 cm (Table 1). Most tumors were small (mean <8 cm, Table 1). In recent years, occasional large adrenal lipomas have been reported. Sharma was the first to describe a symptomatic adrenal lipoma, 12 cm in size and 225 g in weight [6]. Singaporewalla recently reported a 15.6 cm tumor [6]. The size of the lipoma in our patient was 16 × 14 × 7 cm with a weight of 950 g; the only larger one reported was asymptomatic [10]. Finally, 66% of adrenal lipomas develop in the right adrenal gland, whereas in our case the tumor developed in the left adrenal gland (Table 1).

## Conclusion

Lipomas may rarely be found in the adrenal gland. They are usually asymptomatic but occasionally manifest with acute complications such as hemorrhage or infection. This is the first report of an adrenal lipoma presenting with chronic symptoms of mild postprandial abdominal pain and fullness. Our lipoma is the second largest reported in the literature to date. Physicians of all medical specialties should be aware that the differential diagnosis of mild chronic abdominal pain in patients repeatedly presenting to emergency rooms may include large adrenal lipomas. We recommend that these patients, in whom initial diagnostic investigation is unrevealing, merit further investigation. This holds especially true when empiric treatment is unsuccessful. Such patients should be referred to an out-patient specialist clinic for further clinical and laboratory investigation, including abdominal CT scans.

## Consent

Written informed consent was obtained from the patient for publication of this case report and any accompanying images. A copy of the written consent is available for review by the Editor-in-Chief of this journal.

## Competing interests

The authors declare that they have no competing interests.

## Authors' contributions

SK and AT made the diagnosis and performed the adrenalectomy. ID and SK evaluated the medical literature and prepared the manuscript. AF interpreted the histology and edited the final manuscript before submission. All authors read and approved the final manuscript.
